# Development and validation of an automated evaluation system for lattice radiotherapy using a commercial treatment planning system scripting API

**DOI:** 10.1002/acm2.70700

**Published:** 2026-07-27

**Authors:** Xingliu Wang, Ruifeng Zhao, Yongqiang Li, Qinghua Xu

**Affiliations:** ^1^ Department of Radiation Oncology The Second Affiliated Hospital of Nanchang University Nanchang China; ^2^ Department of Radiation Oncology School of Medicine Shanghai Pulmonary Hospital Tongji University Shanghai China; ^3^ Department of Medical Physics Shanghai Proton and Heavy Ion Center Fudan University Cancer Hospital Shanghai China

**Keywords:** dosimetric evaluation, Eclipse scripting API, lattice radiotherapy, spatially fractionated radiation therapy, treatment planning system

## Abstract

**Purpose:**

Clinical implementation of lattice radiotherapy (LRT) remains constrained by the absence of standardized, automated tools for comprehensive post‐plan dosimetric analysis within commercial treatment planning systems (TPS). Herein, an automated script‐based tool was developed using the Eclipse Scripting API (ESAPI) to enable rapid, standardized calculation of key LRT‐specific metrics, including peak‐to‐valley dose ratio (PVDR), equivalent uniform dose (EUD), and ablative dose ratio (ADR).

**Methods:**

A modular computational architecture comprising four functional layers was implemented: (1) user interface for protocol configuration, (2) computation layer integrating multiple PVDR algorithms, (3) data service layer providing direct read‐only access to three‐dimensional dose and anatomical data via ESAPI, and (4) reporting module. To accommodate ESAPI security restrictions, a dual‐script architecture incorporating a preprocessing sphere segmentation module was established. Validation was performed using a retrospective cohort of 12 lung cancer LRT patients, with accuracy assessed against manual TPS measurements and established reference values.

**Results:**

Concordance between automated and manual TPS measurements was high. Standard dose‐volume parameter extraction exhibited perfect agreement. For spatially explicit PVDR analysis, mean absolute error relative to manual profile assessment was 0.034 (absolute VPDR units). EUD calculations deviated less than 3% from published reference data. Evaluation time per plan was reduced from 15.41 ± 3.20 min (manual) to 1.97 ± 0.34 min (automated), representing a 7.8‐fold efficiency gain with elimination of inter‐operator variability.

**Conclusion:**

This ESAPI‐based tool automates and standardizes dosimetric evaluation of LRT, addressing a practical workflow bottleneck. Integration of multiple calculation methods within a deterministic framework establishes a shareable benchmark for consistent multi‐institutional analysis, providing a foundation for future investigations correlating detailed dosimetric parameters with clinical outcomes in spatially fractionated radiotherapy.

## 1 INTRODUCTION

**FIGURE 1:**
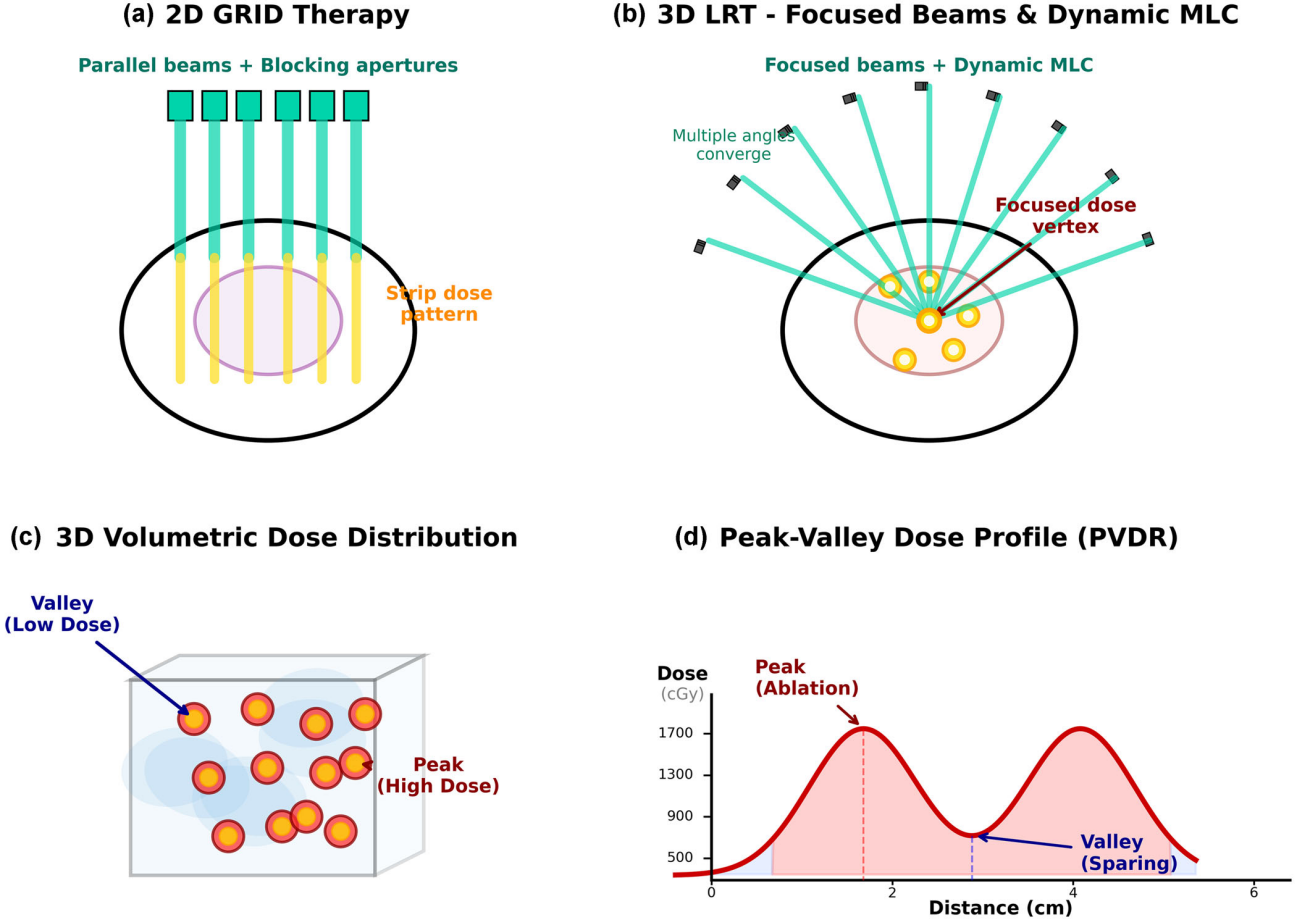
Schematic illustration of lattice radiotherapy (LRT). (a) Conventional two‐dimensional GRID therapy uses parallel beams with blocking apertures to create a strip dose pattern. (b) In contrast, three‐dimensional LRT employs multiple focused beams with dynamic multi‐leaf collimators (MLC) to create discrete high‐dose vertices. (c) The resulting three‐dimensional dose distribution comprises peak regions of high ablative dose separated by valley regions of low sparing dose. (d) An idealized peak‐valley dose profile illustrates the peak‐to‐valley dose ratio (PVDR), defined as the ratio of peak dose to valley dose between adjacent vertices.

Spatially fractionated radiation therapy (SFRT) marks a fundamental departure from the homogeneous dose paradigm that has dominated radiation oncology for decades. This technique shows unique therapeutic promise for managing bulky, recurrent, or radioresistant tumors.^1^ Lattice radiotherapy (LRT)—the three‐dimensional implementation of SFRT—achieves physical dose fractionation through a strategic arrangement of discrete high‐dose “peaks” interspersed with low‐dose “valleys.^2^” In the present study, LRT refers specifically to a three‐dimensional, multi‐beam delivery technique utilizing dynamic multi‐leaf collimators (MLC) to modulate photon beams and create discrete, high‐dose vertices within the tumor volume (Figure 1). Unlike two‐dimensional GRID therapy, which employs physical blocks or static apertures to produce a regular pattern of high‐ and low‐dose regions, LRT distributes these vertices throughout the three‐dimensional tumor volume, enabling more conformal dose sculpting. The therapeutic aim is to deliver ablative doses at peak regions while maintaining low‐dose valleys that spare normal tissue and potentially stimulate systemic anti‐tumor immune responses. Emerging evidence indicates that such extreme intratumoral dose heterogeneity induces direct cytotoxicity while potentially eliciting radiation‐induced bystander effects, remodeling the tumor microenvironment, and stimulating systemic anti‐tumor immunity. Collectively, these effects are believed to explain the favorable tumor response and palliative outcomes observed in patients with bulky or recurrent tumors that respond poorly to conventional radiation.^3^

Although preliminary clinical evidence supports encouraging tumor responses and acceptable toxicity,^4^, ^5^ the broader adoption of LRT faces substantial technical challenges. Current commercial treatment planning systems (TPS), such as Varian Eclipse, are not equipped with intrinsic, standardized tools to perform automated quantitative evaluation of the highly non‐uniform dose distributions characteristic of LRT.^6^ Native TPS plan evaluation tools, such as dose‐volume histograms (DVHs), are not designed to compute core LRT heterogeneity indices. This limitation is compounded by a notable lack of consensus in the literature regarding the precise definition and calculation methodology for these key indices, creating substantial barriers to standardized clinical implementation and cross‐institutional validation.

Take the peak‐to‐valley dose ratio (PVDR), or its inverse, the valley‐to‐peak dose ratio (VPDR), as a key example. Multiple competing definitions coexist, thereby preventing direct cross‐study comparisons: (1) The mean dose ratio (PVDR_MeanRatio_) represents the ratio of mean dose in high‐dose lattice structures to mean dose of the entire gross tumor volume (GTV), offering an efficient global assessment but sacrificing spatial specificity.^7^ (2) The D10/D90 ratio (PVDR_D10/D90_), calculated from the GTV's DVH as the dose covering 10% of volume divided by that covering 90%,^8^, ^9^, ^10^ is widely recommended for characterizing overall heterogeneity though it may inadequately capture focal peak doses.^11^ (3) The valley‐to‐peak dose ratio (VPDR) proposed by Wu et al.^2^ defines the ratio of mean dose in the lowest 5% dose region of the lattice volume to the prescribed peak dose, emphasizing the relationship between valley doses and prescription. (4) A spatially explicit approach calculates the ratio of D1% (peak) dose to dose at the geometric midpoint between adjacent vertices, reporting the median (VPDR_median_).^11^ While this method precisely reflects local gradients, manual implementation proves prohibitively labor‐intensive. Other key evaluation metrics, including the ablative dose ratio (ADR)^7^ and the linear‐quadratic model‐based equivalent uniform dose (EUD),^9^, ^12^ similarly lack standardized TPS‐based calculation tools.

This confluence of definitional ambiguity and computational complexity impedes cross‐institutional outcome comparison and robust protocol development. Consequently, development of an automated computational tool represents a practical step toward standardization in the field. Such a tool must integrate multiple established calculation methods, minimize planner‐dependent subjectivity, and enable efficient, standardized plan evaluation.

To address this need, we developed an automated dose evaluation tool for LRT using the Eclipse Scripting Application Programming Interface (ESAPI). The tool integrates directly into the clinical Eclipse workflow, automatically extracting 3D dose data and computing key heterogeneity indices according to the various established definitions of PVDR and VPDR. Building upon automated VPDR_median_ calculation, we further proposed and implemented a refined variant (PVDR_median‐opt_) that employs dense sampling along lines connecting vertices to better characterize valley dose distributions. This manuscript details the tool's architecture, core algorithms, functional modules, and its application to test cases.

## 2 METHODS

### 2.1 Tool development and system architecture

**FIGURE 2:**
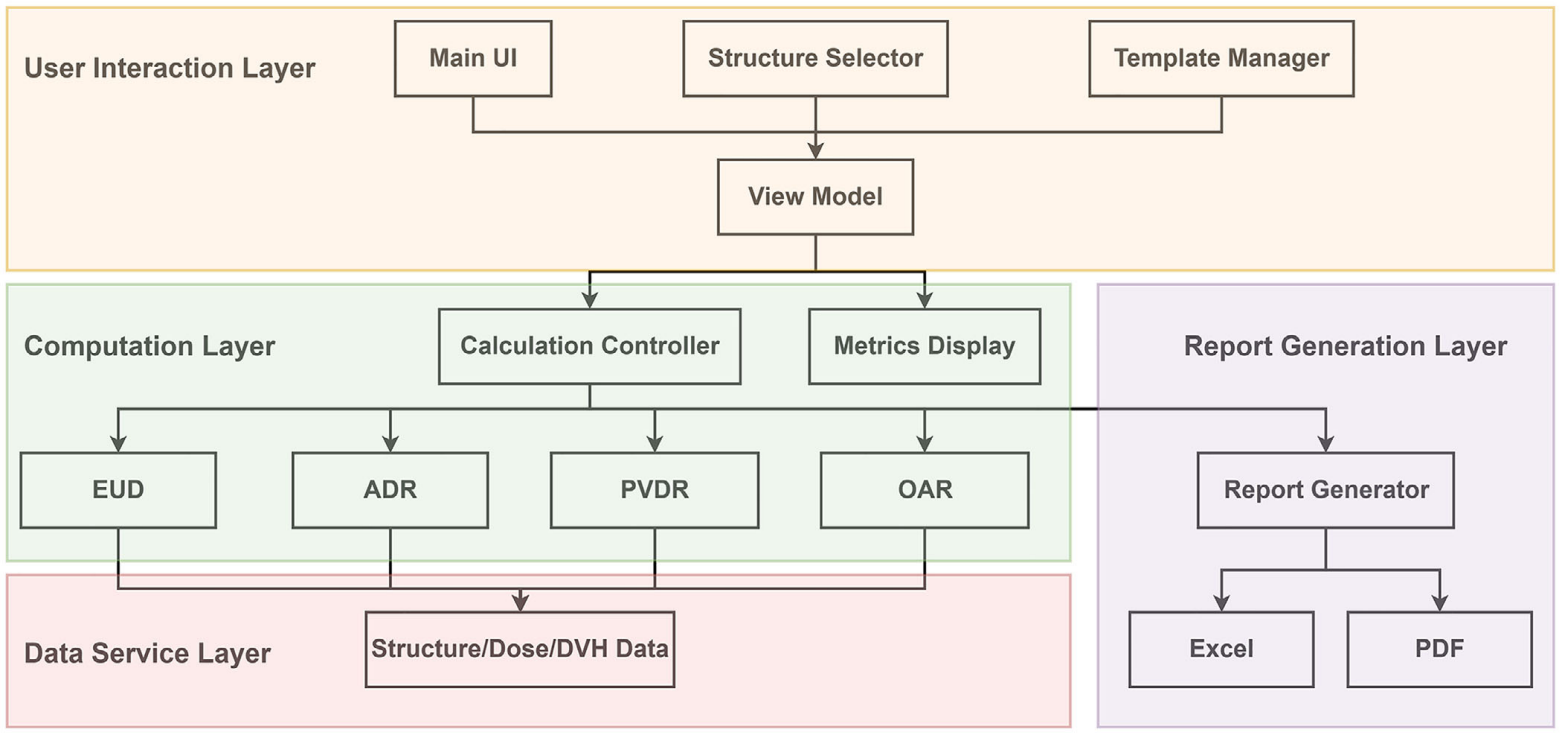
System architecture of the automated Lattice Radiotherapy (LRT) evaluation tool, illustrating the four‐layer design: user interaction, computation layer, data service, and report generation.

An automated, plugin‐based evaluation tool was developed using the Eclipse Scripting API (ESAPI, version 16.1) to facilitate and standardize the dosimetric evaluation of LRT. The tool's architecture was designed as a four‐layer system to achieve full automation, including the user interaction, core computation, data service, and report generation layers (Figure 2).

**FIGURE 3:**
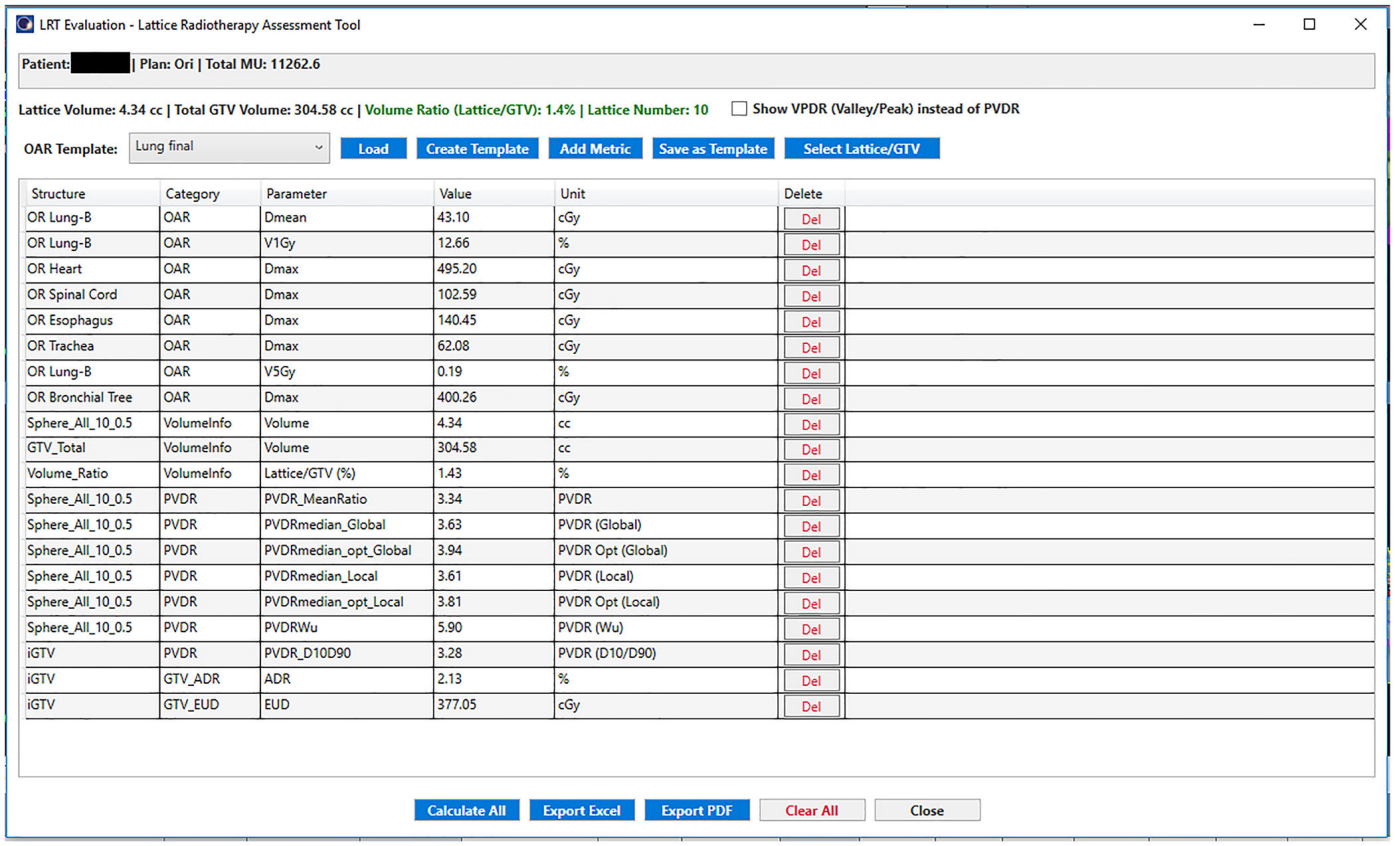
Main graphical user interface of the LRT evaluation tool, showing patient/plan information, configurable parameters, and results panel.

1. User Interaction Layer: This layer provides a graphical user interface (GUI) designed for clinical use by medical physicists (Figure 3). It serves as the entry point for the ESAPI plugin execution. The main interface integrates all evaluation functions and displays results. It enables the rapid configuration of key parameters like α/β values. To accommodate variations in institutional structure‐naming conventions, users can directly designate the relevant structures within the GUI. This layer also allows users to save institution‐specific OAR evaluation criteria as XML templates, promoting protocol uniformity and facilitating team sharing.
2. Computation Layer: This layer serves as the analytical core, encapsulating the calculation logic for key LRT dosimetric indices. The tool integrates multiple evaluation metrics, including various definitions of the PVDR and VPDR, the EUD, and the ADR. For PVDR/VPDR calculation, several methods documented in the literature were implemented. All computations are performed automatically, with required data (3D dose distributions and structure geometries) being read directly from the treatment planning system to minimize human error and ensure reproducibility.
3. Data Service Layer: This layer functions as a secure data bridge, providing real‐time, read‐only access to the TPS database. 3D dose grids, structure geometries, and dose‐volume histogram (DVH) data are retrieved directly from the Eclipse TPS via native ESAPI interfaces. This direct connection eliminates the time overhead and potential errors associated with DICOM file transfers, while ensuring a unidirectional (read‐only) data flow that complies with clinical safety standards.
4. Report Generation Layer: This layer exports the computational results as Excel spreadsheets or PDF documents for clinical documentation, local review, and archiving. The exported reports contain all evaluation indices, monitor unit (MU) data, and relevant patient information. Additionally, results are displayed in the main GUI for immediate review by the physicist. Asynchronous processing was implemented to prevent GUI blocking during computational‐intensive operations.

### 2.2 Core algorithm implementation

#### 2.2.1 Spatial adjacency identification algorithm

Calculation of spatially explicit indices like PVDR_median_ requires accurate identification of geometrically adjacent vertices within the lattice structure. The concept of adjacency is not uniquely defined in LRT: it can be operationalized either by a population‐level distance threshold that references the collective geometry of all vertices, or by a local geometric criterion that evaluates each candidate pair independently against its immediate neighborhood. We therefore implemented two complementary adjacency modes—Global and Local—grounded in these two distinct philosophies. Both modes operate on the same vertex spatial layout but apply different decision rules, enabling users to assess how the choice of adjacency definition influences the resulting dosimetric indices.

Global mode employs an adaptive dynamic threshold based on the intrinsic spatial distribution of the vertex set. This approach addresses a key limitation of fixed‐distance thresholds (e.g., 20–40 mm), which exhibit suboptimal performance across varying tumor sizes and lattice densities by failing to adapt to patient‐specific geometries. The algorithm first calculates *D_median_*, the median of all inter‐centroid distances within the vertex population. A dynamic threshold *T* is subsequently defined as *T = k × D_median_*, where *k* represents an empirical coefficient set to 1.3. This coefficient was validated across a patient cohort and was found to balance sensitivity and specificity for adjacency recognition across typical clinical geometries. The threshold is constrained to the range [8 mm, 45 mm], where the lower bound prevents collapse to unphysically small values at very low *k* and the upper bound captures face‐diagonal connections (∼42 mm for 30‐mm CTC spacing) while excluding body‐diagonal pairs (∼52 mm). The workflow involves extracting vertex centroids, constructing a Euclidean distance matrix, determining *D_median_*, and computing the adaptive threshold. Pairs with inter‐centroid distance ≤ *T* × 1.02 are classified as adjacent, where the 2% margin accounts for minor geometric uncertainties. The stability and generalizability of *k* = 1.3 are further discussed in Appendix A.

Local mode constructs the adjacency graph using the Gabriel graph,^12^ a parameter‐free geometric structure that evaluates each candidate pair strictly against its local neighborhood. A vertex pair (i, j) is considered adjacent if and only if no third vertex *k* exists such that $d_{ik}^{2}+d_{jk}^{2}<d_{ij}^{2}$, where *d* denotes the Euclidean inter‐centroid distance. Equivalently, the open sphere with diameter equal to the inter‐vertex segment must contain no other vertex centroids. This criterion ensures that only “direct” neighbors—those with an unobstructed line of sight in the local geometric sense—are admitted. By evaluating each pair independently of population‐level statistics, Local mode tends to produce sparser adjacency graphs. It excludes face‐diagonal connections that Global mode may include when the population *D_median_* is large, because such pairs typically fail the Gabriel condition when an intermediate vertex exists. The computational implementation operates entirely on the pairwise distance matrix, making it dimension‐independent and numerically stable. A systematic comparison of the two modes across 29 configurations is presented in Appendix A.

#### 2.2.2 Multi‐mode PVDR calculation framework

We developed an integrated multi‐mode framework for automatic computation of established PVDR/VPDR indices:

1. PVDR_MeanRatio_: The script automatically retrieves DVH data for the high‐dose lattice structure (GTV_Lattice) and the gross tumor volume (GTV), calculates their mean doses, and computes the ratio. This mode offers high computational efficiency, facilitating rapid plan assessment.
2. PVDR_D10/D90_: The tool automatically extracts the GTV's DVH curve, identifies the doses covering 10% (D_10%_) and 90% (D_90%_) of the volume, and calculates their ratio. This index is recommended by multiple publications and is regarded as a robust parameter for characterizing overall dose heterogeneity.
3. VPDR per Wu et al.^2^: The script calculates the mean dose within the lowest 5% dose region of the lattice volume (V_L_) (D_mean(95‐100)_) and divides it by the prescribed peak dose (D_p_) to obtain the VPDR value.
4. Spatially Explicit VPDR_median_: This method provides high spatial precision for characterizing local dose gradients. The algorithm first uses adjacency algorithm (Global or Local) to identify all spatially adjacent vertex pairs. For each pair, the system automatically extracts the D1% dose (peak) and the dose at the geometric midpoint of the line connecting the two centroids (valley). The individual valley‐to‐peak ratio (VPDRij) is calculated for each pair, and the final reported value is the median of all ratios. This implementation fully automates the calculation logic described by Prado et al.,^11^ thereby transforming a traditionally labor‐intensive manual process into an efficient, automated workflow.
5. PVDR_median‐opt_ (our refined variant of the standard VPDR_median_ method): This method follows the same adjacency identification and peak extraction as the standard VPDR_median_. The refinement lies in the valley dose sampling strategy: rather than using a single midpoint dose, this method performs high‐density linear sampling along the line connecting the centroids of adjacent vertices. This refinement improves the spatial sampling resolution along the peak‐valley axis to approximately 1 mm, although its incremental clinical significance relative to the standard midpoint approach remains to be determined.

All calculations access 3D dose grids and structural data directly through ESAPI interfaces, which ensures accuracy and reproducibility. All established PVDR/VPDR calculation modes are executed automatically upon initiation, with computational processing completed within approximately 30–60 s on a standard Eclipse physicist workstation. The reciprocal mathematical relationship between VPDR and PVDR is accommodated to allow quick display toggle between these related indices via the GUI.

#### 2.2.3 Automated extraction of the ablative dose ratio (ADR)

The ablative dose ratio (ADR), defined as the percentage of the target volume receiving at least the prescription dose, represents a key index for LRT plan evaluation.^7^ Our tool automatically reads the prescription dose from the loaded treatment plan. Using ESAPI methods, it retrieves the absolute volume within the target structure that satisfies this dose threshold (i.e., volume receiving ≥ prescription dose), subsequently calculating the corresponding percentage of the total target volume. This fully automated process requires no manual input, thereby minimizing potential operator error. The reported results include both the absolute ablated volume within the GTV (V_GTV_Abl_) and the GTV volume (V_GTV_) to facilitate physicist verification.

#### 2.2.4 Numerically stable implementation of equivalent uniform dose (EUD)

The equivalent uniform dose (EUD), based on the linear‐quadratic (LQ) model, constitutes a key parameter for evaluating the biological effectiveness of non‐uniform dose distributions. It is calculated using the established formula,^9^, ^13^

(1) $$EUD=-\frac{\alpha }{2\beta }+\frac{1}{2}{\left[{\left(\frac{\alpha }{\beta }\right)}^{2}-\frac{4}{\beta }\ln\left(\sum_{j}v_{j}e^{-\alpha D_{j}-\beta D_{j}^{2}}\right)\right]}^{\frac{1}{2}},$$

where D_j_ is the dose deposited in sub‐volume v_j_, and α and β are radiosensitivity parameters. To ensure robust numerical stability when processing clinical dose distributions, the algorithm incorporates multiple safeguards: (1) overflow protection for exponential terms involving very high doses (where resultant negligible values are skipped), (2) implementation of the Kahan summation algorithm to mitigate floating‐point accumulation errors during the summation of exponential terms, and (3) validation of the argument for the square root operation prior to the final calculation to guarantee the output is a clinically meaningful real number.

### 2.3 Lattice structure preprocessing module

In clinical LRT practice, multiple lattice vertices are often stored as a single aggregated structure (e.g., GTV_Lattice). While efficient for planning purposes, this aggregated format obstructs access to individual sphere dose‐volume data during subsequent evaluation, thereby preventing the automated calculation of spatially explicit PVDR indices. Ideally, a fully integrated workflow would perform structure segmentation directly within the evaluation tool. However, the ESAPI sandbox security model presents a limitation: scripts operate in a read‐only mode after initialization, preventing write operations like creating new structures during runtime.

To circumvent this technical constraint, we implemented a loosely coupled, dual‐script architecture. A dedicated preprocessing script (Sphere_Creator) handles structure management and writing. The core evaluation script (LRT_Evaluation) maintains a read‐only status to ensure operational security and stability. The preprocessor outputs individually segmented spheres following the “Sphere_X” naming convention, which the main evaluation script automatically identifies and loads via name‐prefix matching. Communication between the two modules is achieved indirectly through the TPS database, adhering to ESAPI security protocols. This design respects system security, maintains a clear separation of responsibilities, and facilitates independent maintenance, albeit introducing an additional manual step.

The preprocessor employs a surface geometry clustering strategy. It extracts all vertex coordinates from the 3D surface mesh of the aggregated structure to form a point cloud. The Density‐Based Spatial Clustering of Applications with Noise (DBSCAN) algorithm is then applied to automatically identify individual spheres based on spatial point density. Densely connected regions are classified as candidate spheres. The algorithm incorporates two key optimizations: (1) spatial hashing indices to dramatically improve computational efficiency, reducing processing time for cases with numerous spheres to under one min; (2) intelligent post‐processing to filter isolated noise points and merge unrealistically close clusters, preventing erroneous splitting of a single sphere. The algorithm relies on two intuitive, clinically relevant parameters: a neighborhood radius (ε, default 3 mm) defining the maximum distance between two surface points for them to be considered neighbors in the clustering process, and a minimum points (minPts, default 8) specifying the minimum number of neighboring points required to classify a point as part of a dense cluster (i.e., a genuine sphere) rather than noise. Points satisfying both criteria are grouped together. For typical clinical configurations where lattice vertices have diameters of approximately 1 cm and center‐to‐center spacing of approximately 3 cm, these defaults ensure that points belonging to the same spherical surface are grouped together, while points from adjacent vertices or artifacts are excluded.

## 3 RESULTS

### 3.1 Validation of dosimetric accuracy and core algorithm performance

The automated evaluation tool exhibited high accuracy and reliability for all calculated dosimetric parameters. Automated extraction of fundamental parameters (e.g., mean OAR doses and volumes receiving specific dose levels) showed perfect concordance with manual queries performed directly within the TPS, reflecting direct retrieval of identical pre‐computed data via native ESAPI interfaces, thereby confirmed error‐free data retrieval and basic calculations.

For PVDR/VPDR metrics, the tool accurately computed all established definitions. Accuracy for DVH‐based methods (PVDR_MeanRatio_ and PVDR_D10/D90_) was inherent owing to validated baseline data extraction. For the VPDR definition proposed by Wu et al., the script‐derived results exhibited concordance with manual DVH analysis. For the spatially explicit methods (VPDR_median_ and VPDR_median‐opt_), validation using simplified cases containing two or three idealized spheres demonstrated excellent agreement. The mean absolute error (MAE) between the automatically calculated median VPDR for adjacent sphere pairs and the results from manual TPS profile analysis was 0.034 (absolute VPDR units).

Validation of the EUD calculation focused on reproducibility of published results. Using specific cases from the literature with fully reported parameters and corresponding EUD values, EUD was recalculated by the script using identical α/β ratios and prescription doses. For instance, for an LRT configuration (vertex diameter: 1.5 cm, CTC: 3.5 cm), our script calculated a GTV EUD of 3.4 Gy for a case with a GTV volume of 354.25 cm^3^. This result agreed closely with the literature‐reported median EUD of 3.5 Gy for that configuration, deviating by less than 3% and thereby confirming consistency with established methods.^9^

### 3.2 Clinical workflow validation

The tool's clinical applicability was evaluated in a retrospective study of 12 lung cancer patients treated with LRT. All patients were simulated with four‐dimensional computed tomography (4D‐CT), and treatment plans were generated using internal target volume (ITV)‐based techniques with lattice vertices positioned within an ITV inward margin (typically 8–10 mm) to ensure geometric coverage throughout the breathing cycle. A single fraction of 12 Gy or 15 Gy was prescribed to the lattice vertices. Patient and treatment characteristics are summarized in Table 1.

**TABLE 1:** Patient characteristics (*n* = 12).

Patient	ITV volume (cm^3^)	Prescription dose (Gy)	Number of vertices	Median CTC (mm)
1	352.54	12	7	29.1
2	237.03	12	7	29.4
3	169.70	12	3	30.0
4	304.58	12	8	28.5
5	354.25	12	6	29.7
6	137.34	15	2	28.4
7	137.88	12	3	25.7
8	322.64	12	8	29.4
9	221.56	12	5	28.0
10	212.52	15	5	29.2
11	192.55	15	4	29.2
12	217.97	15	4	29.8

*Note*: Median CTC denotes the median inter‐centroid distance of adjacent vertex pairs identified by the Gabriel graph (Local mode), reflecting the actual geometric configuration.

**FIGURE 4:**
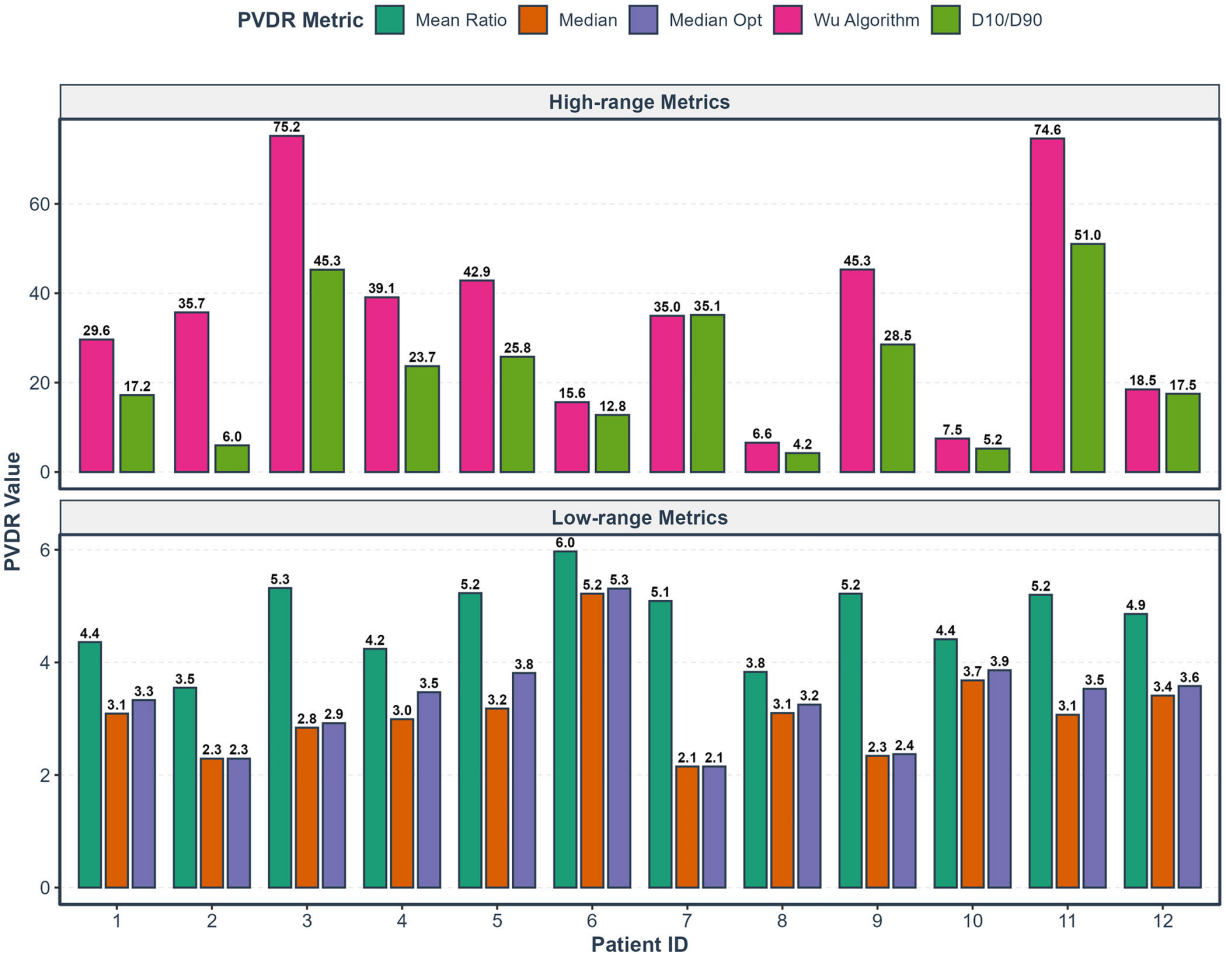
Comparison of five different PVDR calculation methods across 12 lung cancer LRT cases. The spatially explicit methods (PVDR_median_ and PVDR_median‐opt_) were computed using Global adjacency mode.

A comparative summary of the PVDR results from the five different calculation modes for this cohort is presented in Figure 4. The two spatially explicit methods (PVDR_median_ and PVDR_median‐opt_) were both computed using Global adjacency mode; Local mode was simultaneously calculated and produced comparable results for cases with *N* ≤ 6, with modest divergence observed at *N* = 7–8, consistent with the expected equivalence for small lattice sizes demonstrated in Appendix A. Based on their numerical magnitude and underlying calculation methodology, the five methods clustered into two distinct groups: a high‐range group comprising methods that yield large absolute ratios (PVDR_Wu_ and PVDR_D10/D90_), and a low‐range group comprising methods with compressed distributions (PVDR_MeanRatio_, PVDR_median_, and PVDR_median‐opt_). PVDR_Wu_ and PVDR_D10/D90_ constituted a high‐range group, with mean values of 35.46 and 22.70, respectively, exceeding the other methods by approximately one to two orders of magnitude. In contrast, PVDR_MeanRatio_, PVDR_median_, and PVDR_median‐opt_ formed a low‐range group exhibiting compressed distributions (mean values: 4.77, 3.11, and 3.31, respectively; all maxima < 6.0).

Substantial inter‐patient heterogeneity was observed within the high‐range group, with values for PVDR_Wu_, for example, ranging from 6.56 to 75.20. The low‐range group displayed relatively bounded variations, with PVDR_MeanRatio_ showing the most constrained spread (mean ± SD: 4.77 ± 0.70, range: 3.55 – 5.97). Notably, our refined median method (PVDR_median‐opt_) consistently yielded marginally higher values than the standard PVDR_median_ for all patients, reflecting the more granular spatial sampling along the peak‐valley axis. The incremental clinical significance of this difference relative to the standard midpoint approach warrants further prospective validation.

A substantial practical benefit was the marked improvement in evaluation efficiency. Statistical analysis of the 12 cases revealed that the comprehensive dosimetric evaluation time per case (including plan loading, computation, result review, and report generation; excluding the manually impractical EUD calculation) was reduced from 15.41 ± 3.20 min (manual) to 1.97 ± 0.34 min (automated), representing a 7.8‐fold increase in efficiency. This time reduction translates into tangible clinical workflow benefits: it alleviates physicist workload during plan evaluation, shortens planning timeline delays by reducing the evaluation bottleneck, decreases decision latency by enabling near‐real‐time evaluation feedback, and unlocks large‐scale retrospective analyses previously impractical due to labor intensity.

### 3.3 Validation of structure preprocessing accuracy

The geometric fidelity of the SphereCreator preprocessing script constitutes a key requirement for accurate subsequent analysis. Using voxel‐level Boolean operations, clinically common aggregated lattice structures were successfully segmented into their constituent individual spheres. Validation across 12 test structures demonstrated high segmentation accuracy. The relative volume difference (*δV*) between the summed volume of all segmented spheres and the original aggregated structure exhibited a median of 0.0%. In 75% of cases (9/12), the absolute δV was less than 2%. Across all cases, *δV* ranged from ‐5.3% to 0.0%, with three cases showing mild volume underestimation between 2% and 5% (*δV* = ‐3.3%, ‐4.0%, ‐5.3%). Importantly, the absolute volume discrepancy in these cases was negligible (< 0.1 cm^3^). These results indicate that the segmentation process introduced no significant geometric distortion; the observed minor differences are attributable to inherent volume‐calculation and sampling precision limitations of the TPS. Thus, the script provides a reliable structural foundation for spatial lattice dose analysis.

## 4 DISCUSSION

We developed and validated an ESAPI‐based tool for the automated calculation of key SFRT/LRT dosimetric indices. Its primary value lies in offering a practical solution to the standardization gap in plan evaluation, which constitutes a major bottleneck hindering the translation of LRT from exploratory research to routine clinical practice. While current research primarily focuses on automating lattice vertex placement or plan design (i.e., automated generation of LRT plans),^9^, ^14^, ^15^, ^16^, ^17^ our work addresses automation of post‐plan evaluation (i.e., consistent and efficient evaluation of existing LRT plans). Accordingly, this tool serves as a complementary component necessary for a complete technical workflow, spanning from plan generation to quality assessment.

The central challenge in the clinical adoption of LRT is the lack of standardized dosimetric evaluation, stemming from two intertwined factors. First, the radiobiological mechanisms underpinning LRT's unique efficacy are not fully elucidated, rendering the derivation of optimal physical parameters from first principles difficult.^18^ Second, no consensus exists regarding the definition and calculation of key indices such as PVDR/VPDR. The literature reveals a proliferation of definitions, with studies using mean‐dose ratios, DVH‐based ratios, or spatially explicit methods.^2^, ^7^, ^9^, ^11^ This inconsistency prevents the direct comparison of dosimetric data across studies, thereby severely impeding multi‐institutional collaboration and formation of clinical consensus.

In this context, the principal contribution of this tool is the transformation of disparate theoretical definitions into an executable, deterministic, and standardized operational framework. Adopting a pragmatic strategy of inclusive integration, multiple mainstream calculation algorithms are incorporated and automated within a single interface. This capability enables different institutions to perform post‐planning dosimetric evaluations using a common computational logic, thereby establishing a shared dosimetric language for future research. This represents a direct response to calls for standardized data reporting in SFRT trials.^19^, ^20^

Furthermore, the tool achieves an order‐of‐magnitude improvement in evaluation efficiency. In the present validation, the single‐case evaluation time was reduced from an average of 15.41 min to 1.97 min. This efficiency gain is substantial and clinically meaningful. It enables large‐scale retrospective analyses and the feasible centralized processing of multi‐center trial data. Previously, the tedious nature of manually calculating indices such as VPDR_median_ likely compelled studies to avoid these precise metrics or to use simplified proxies, potentially sacrificing important spatial information. The automated implementation described herein overcomes this barrier. Beyond time savings, the automation eliminates operator‐dependent variability inherent in manual profile placement and point selection, thereby improving reproducibility across users and institutions. This efficiency unlocks the potential to explore correlations between detailed dosimetric parameters and clinical outcomes, which constitutes an important step for translational research.

A further consideration concerns the adjacency algorithm. Global mode and Local mode represent two valid but distinct strategies for constructing the adjacency graph from the same vertex spatial layout. Global mode, with its population‐level threshold, tends to produce more inclusive adjacency graphs that can lead to higher PVDR_median_ estimates in complex configurations. Local mode, with its strictly geometric criterion, produces sparser graphs that can lead to lower PVDR_median_ estimates. The two modes diverge meaningfully for vertex counts *N* ≥ 7, where Local mode detects 10–15% fewer adjacent pairs. Although the majority of cases in this clinical cohort (*N* ≤ 6) fell within the range of equivalence, with modest divergence observed at *N* = 7–8, future work should evaluate the clinical implications of this divergence in larger lattice configurations. The availability of both modes within the same tool—alongside MeanRatio, D10/D90, and Wu algorithm calculations—enables users to compare multiple dosimetric perspectives without additional implementation burden, supporting both routine clinical evaluation and exploratory research.

Nevertheless, the role and current limitations of this tool must be clearly defined. It functions as a tool for advancing the field, rather than serving as a final endpoint. Several limitations indicate directions for future work. First, platform dependency constitutes a constraint. Having been developed on Eclipse v16.1, the tool may be affected by potential API variations across different TPS versions, which could limit its stable operation and rapid multi‐center deployment. Second, all validation cases in this retrospective study were planned using 4D‐CT‐based motion management with lattice vertices positioned within an ITV inward margin; the tool itself evaluates only the static dose distribution on the average intensity projection (AIP) CT and does not capture motion‐induced dose smearing. Third, the scope of validation requires expansion. The present verification was limited to a single clinical site and focused on specific tumor sites and lattice patterns, with algorithmic accuracy assessed against manual measurements performed by a single experienced physicist. Formal multi‐observer reproducibility studies and plan‐acceptance decision analyses were beyond the scope of this initial work and represent important directions for future investigation. The robustness of the tool across a wider range of anatomical locations, irregular geometries, or novel lattice placement strategies requires further validation with larger and more diverse datasets. Finally, the depth of integration could be enhanced. Future work could explore deeper coupling with automated plan generation modules, aiming to construct a seamless end‐to‐end workflow from plan design to quality evaluation and reporting.

## 5 CONCLUSION

We developed and validated an automated dosimetric evaluation tool for LRT using the ESAPI. The tool enables direct, high‐fidelity access to 3D dose and structural data. It efficiently computes key clinical indices, including the PVDR/VPDR, EUD, and ADR, within a streamlined workflow. Clinical validation demonstrated a greater than sevenfold improvement in evaluation efficiency while preserving dosimetric accuracy and consistency. This tool addresses the absence of dedicated SFRT evaluation tools in commercial TPS. Beyond accelerating routine assessment, the tool provides a shareable and standardized benchmark framework. It establishes a common dosimetric language for the SFRT community and provides a deterministic technical foundation for future multi‑institutional studies investigating dose‑response relationships in spatially fractionated radiotherapy.

## AUTHOR CONTRIBUTIONS

**Xingliu Wang**: Conceptualization; methodology; software; formal analysis; writing—original draft. **Ruifeng Zhao**: Software validation; technical feedback. **Yongqiang Li**: Workflow review; manuscript feedback. **Qinghua Xu**: Study supervision; results discussion; writing—review and editing.

## CONFLICT OF INTEREST STATEMENT

The authors declare no conflicts of interest.

## ETHICAL STATEMENT

The need for ethics approval was waived for this retrospective study, as all patient data were fully anonymized and de‐identified prior to analysis, and the study was limited to software validation without any clinical intervention or patient contact.

## Supporting information



Supporting Information

## Data Availability

The data from this manuscript will be made available upon reasonable request to the corresponding author.
