# Supplementary material for: Development and validation of an automated evaluation system for lattice radiotherapy using a commercial treatment planning system scripting API
Source: J Appl Clin Med Phys. 2026 Jul 27;27(8):e70700. doi: 10.1002/acm2.70700 (PMC13404247; doi:10.1002/acm2.70700)
Supplement: Supplementary file 1 — Supporting Information [file ACM2-27-e70700-s001.docx]

**APPENDIX A**

**Adjacency Algorithm: Sensitivity of k and Comparison of Global and Local Modes**

# A.1 Introduction

The adjacency algorithm described in the main manuscript (Section 2.2.1) defines two complementary modes for identifying spatially adjacent vertex pairs within a lattice structure: Global mode and Local mode. Both modes construct the adjacency graph required for calculating spatially explicit PVDR_median_, but they differ in their underlying philosophy. Global mode uses a population-level threshold based on an empirical coefficient k, whereas Local mode uses a purely geometric, parameter-free criterion based on the Gabriel graph. This Appendix addresses two independent questions: (1) Is k = 1.3 a robust default for Global mode? (2) How do Global and Local modes differ, and under what conditions can they be used interchangeably?

In Global mode, the effective adjacency threshold is T = k × D_median_, where D_median_ is the median of all inter-centroid distances. The coefficient k = 1.3 is the default presented in the main manuscript. The threshold is constrained to [8 mm, 45 mm]. The lower bound prevents collapse to unphysically small values at very low k; it was never triggered in this cohort. The upper bound captures face-diagonal connections (~42 mm for 30-mm CTC spacing) while excluding body-diagonal pairs (~52 mm), which rarely correspond to clinically relevant dose valleys.

Local mode evaluates each candidate pair strictly against its local geometric context. A vertex pair (i, j) is adjacent if and only if no third vertex k exists such that $d_{ik}^{2}+d_{jk}^{2}<d_{ij}^{2}$, where d denotes the Euclidean inter-centroid distance. This is the defining criterion of the Gabriel graph. Because it evaluates each pair strictly against its local neighborhood rather than against a population-level statistic, Local mode produces sparser adjacency graphs: it excludes face-diagonal connections that the Global threshold captures. The analysis cohort comprised 12 lung cancer LRT patients, yielding 29 vertex configurations with vertex counts ranging from 2 to 15, all treated with a clinically common CTC spacing of approximately 3 cm.

# A.2 Sensitivity Analysis of the Adjacency Coefficient k

## A.2.1 Software Extension for Sensitivity Analysis

The analysis was built upon the LRT_Evaluation script described in the main manuscript (Section 2.2), an ESAPI-based plugin for automated dosimetric evaluation of LRT. The computation layer was extended to support both Global and Local adjacency modes.

The interface of the sensitivity analysis module is shown in Fig. A1. The integrated panel permits systematic batch evaluation of the adjacency detection parameters across all patient configurations.


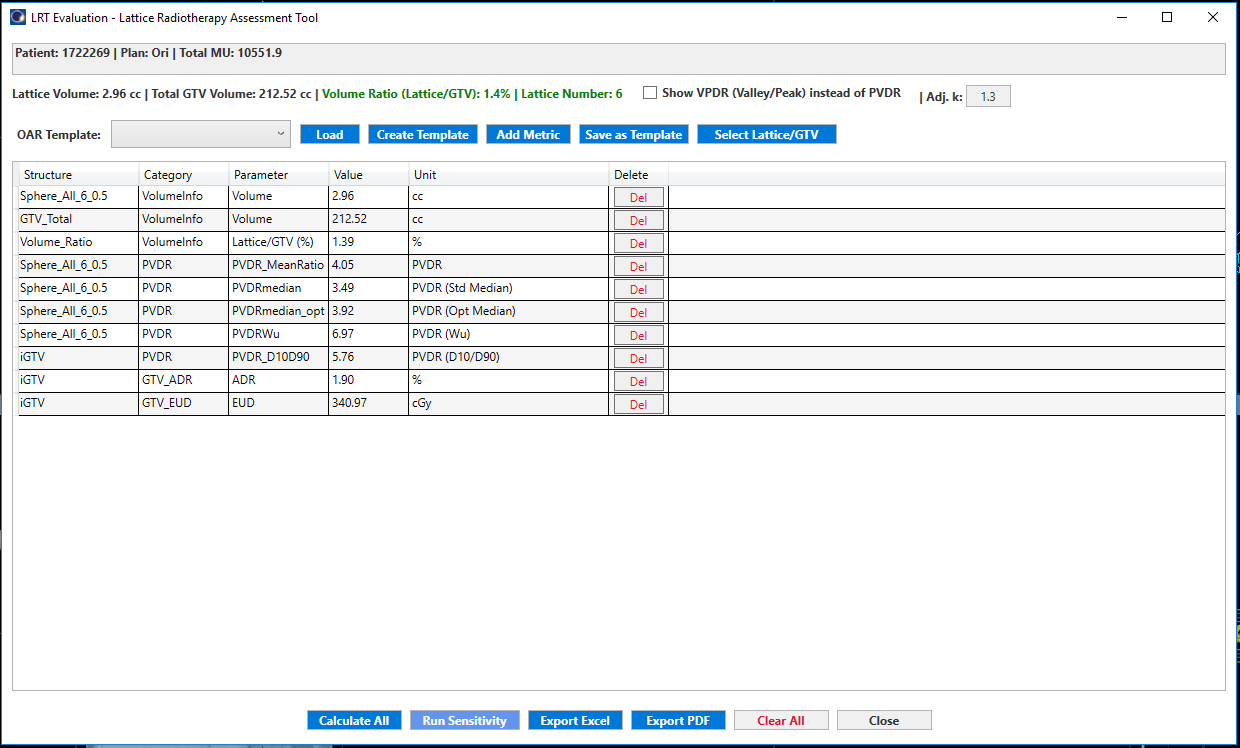


**Fig. A1.** Interface of the sensitivity analysis module integrated into the LRT_Evaluation tool. The panel allows systematic variation of the adjacency coefficient k and selection of fixed-distance thresholds, with automatic computation of Pair Count and PVDR_median_ across all configurations.

## A.2.2 Data Acquisition

Data were acquired retrospectively from 12 lung cancer patients treated with LRT. For each patient, the approved treatment plan was copied within the Eclipse TPS, and alternative lattice configurations were generated by modifying the vertex placement pattern, yielding varying vertex counts (N = 2–15) across 29 total configurations. In clinical practice, vertex placement typically employs an inward margin of 0.8–1.0 cm from the GTV boundary to protect surrounding organs at risk. To increase the number of vertices for sensitivity testing, alternative configurations were generated by reducing the inward margin to 0.3 or 0.5 cm for selected patients.

## A.2.3 Analysis Protocol

Two categories of threshold configurations were evaluated:

(1) Dynamic adaptive thresholds (Global mode). The coefficient k was varied from 0.8 to 2.0 in increments of 0.1. For each k, D_median_ was computed as the median of all pairwise inter-centroid distances. The theoretical threshold T_theory = k × D_median_ was constrained to [8 mm, 45 mm] to yield T_eff (see A.1 for rationale). Adjacent pairs were identified using d_ij ≤ T_eff × 1.02, where the 2% margin accounts for minor geometric uncertainties.

(2) Fixed-distance thresholds. Five fixed thresholds (20, 25, 30, 35, and 40 mm) were evaluated as controls. Pairs with inter-centroid distances below each threshold were considered adjacent.

For each threshold configuration under Global mode, the following metrics were computed: (i) Pair Count: the number of vertex pairs satisfying the adjacency criterion; (ii) PVDR_median_: the median peak-to-valley dose ratio across all detected adjacent pairs, where the peak dose is the mean D1% dose of the two vertices and the valley dose is the dose at the geometric midpoint between their centroids. In parallel, Local mode adjacency graphs were constructed for all 29 configurations using the Gabriel graph criterion.

## A.2.4 Statistical Analysis

For each k value and fixed threshold, aggregate statistics were computed across all 29 configurations, including mean, standard deviation, minimum, and maximum of Pair Count and PVDR_median_. The relative difference in mean pair count between k = 1.3 and each fixed threshold was calculated to quantify the under-detection rate of fixed thresholds.

The saturation point of the sensitivity curve was identified as the smallest k value beyond which the marginal gain in mean pair count fell below 2%. This criterion ensures that the selected k captures the complete adjacency graph without unnecessary expansion.

# A.2.5 Results

## A.2.5.1 Pair Count Stability

Table A1 summarizes how Pair Count and PVDR_median_ vary with k across all 29 configurations. Both metrics exhibit a characteristic saturation curve: rapid rise at low k followed by saturation at higher k.

**Table A1.** Aggregate pair count and PVDR_median_ statistics for dynamic k values (N = 29 configurations).

| k | Pair count | | | | PVDR_median_ | | Configurations with pairs |
| --- | --- | --- | --- | --- | --- | --- | --- |
|  | Mean | SD | Min | Max | Mean | SD |  |
| 0.8 | 9.17 | 12.91 | 0 | 43 | 1.53 | 1.25 | 18/29 |
| 0.9 | 11.83 | 13.31 | 0 | 46 | 2.13 | 0.94 | 25/29 |
| 1.0 | 15.76 | 13.73 | 1 | 53 | 2.85 | 0.69 | 29/29 |
| 1.1 | 16.34 | 13.73 | 1 | 53 | 2.91 | 0.67 | 29/29 |
| 1.2 | 17.00 | 13.40 | 1 | 53 | 3.01 | 0.67 | 29/29 |
| 1.3 | 17.69 | 13.40 | 1 | 53 | 3.10 | 0.61 | 29/29 |
| 1.4 | 17.72 | 13.39 | 1 | 53 | 3.10 | 0.61 | 29/29 |
| 1.5 | 17.86 | 13.30 | 1 | 53 | 3.10 | 0.61 | 29/29 |
| 1.6 | 17.90 | 13.29 | 1 | 53 | 3.10 | 0.62 | 29/29 |
| 1.7 | 17.90 | 13.29 | 1 | 53 | 3.10 | 0.62 | 29/29 |
| 1.8 | 17.90 | 13.29 | 1 | 53 | 3.10 | 0.62 | 29/29 |
| 1.9 | 17.90 | 13.29 | 1 | 53 | 3.10 | 0.62 | 29/29 |
| 2.0 | 17.90 | 13.29 | 1 | 53 | 3.10 | 0.62 | 29/29 |

At k = 0.8, the mean pair count was only 9.17, with 11 of 29 configurations yielding zero adjacent pairs, indicating severe under-detection. As k increased to 1.0, the mean pair count rose to 15.76, and all 29 configurations detected at least one pair, marking the practical lower limit for meaningful adjacency detection. The rising trend continued to k = 1.2 (mean: 17.00) and reached 17.69 at k = 1.3.

Beyond k = 1.3 the marginal gain was negligible (+0.03 pairs, +0.2% from k = 1.3 to 1.4), whereas the transition from k = 1.2 to 1.3 added a meaningful +0.69 pairs. This ~20-fold drop in marginal return identifies k = 1.3 as the saturation point.

Figure A2 presents the per-patient pair count sensitivity to k. Each panel corresponds to one patient, with colored lines distinguishing different vertex counts.


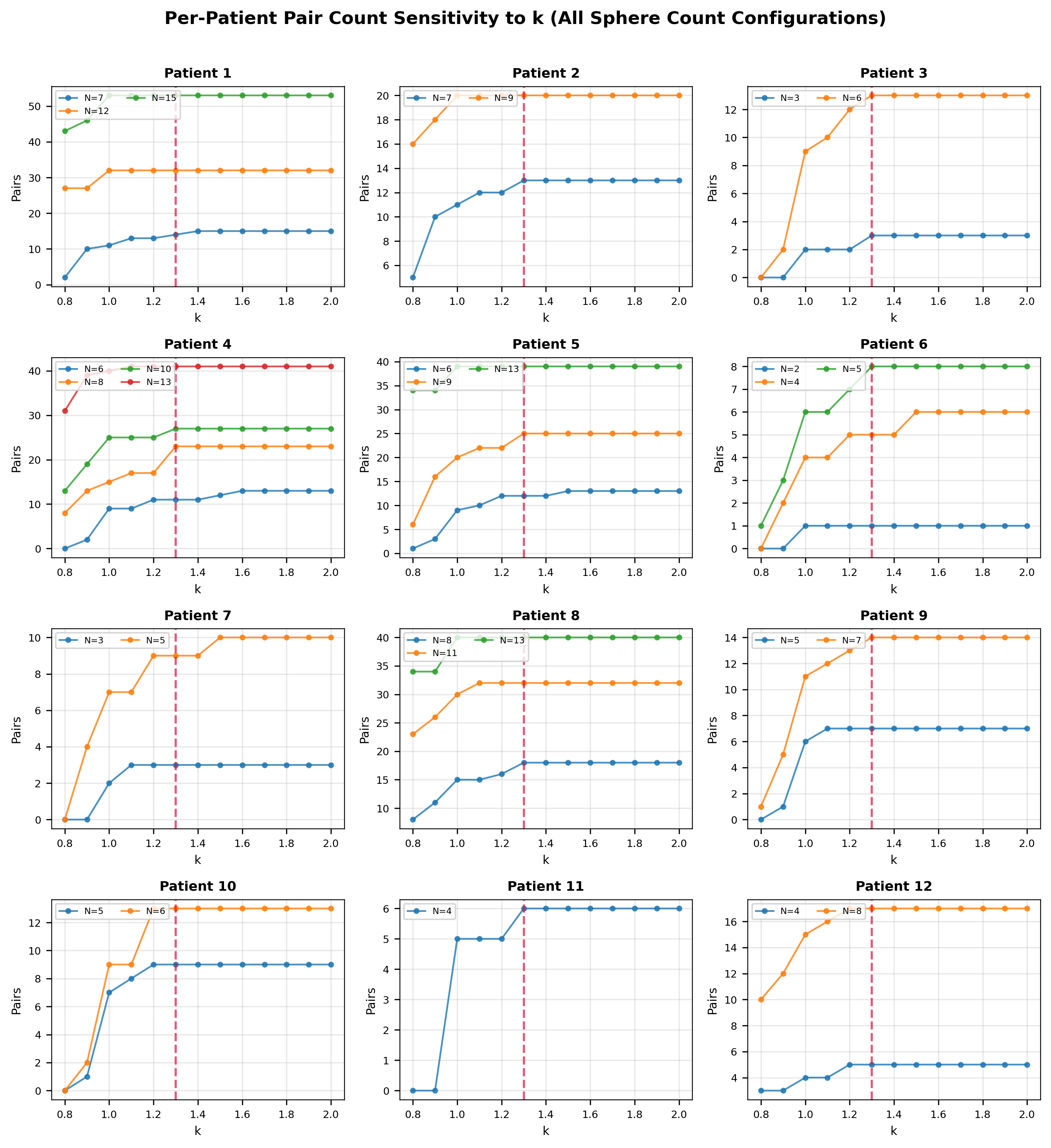


**Fig. A2.** Per-patient pair count sensitivity to k. Each panel represents one patient; colored lines indicate different vertex counts (N). The red dashed line marks k = 1.3.

## A.2.5.2 Comparison with Fixed-Distance Thresholds

We compared k = 1.3 against five fixed thresholds (20–40 mm; Table A2). Fixed thresholds exhibited substantial under-detection across the test range.

**Table A2.** Comparison of fixed-distance thresholds versus adaptive k = 1.3.

| Threshold Strategy | Mean Pairs | Configs with Pairs | Mean PVDR_median_ |
| --- | --- | --- | --- |
| Fixed 20 mm | 0.0 | 0/29 | 0.00 |
| Fixed 25 mm | 0.2 | 6/29 | 0.47 |
| Fixed 30 mm | 11.6 | 29/29 | 2.48 |
| Fixed 35 mm | 14.0 | 29/29 | 2.71 |
| Fixed 40 mm | 15.9 | 29/29 | 2.96 |
| Adaptive k = 1.3 | 17.7 | 29/29 | 3.10 |

For example, Patient 4 (N = 8) produced only 13 pairs at 30 mm versus 23 pairs at k = 1.3. Even the 40 mm threshold detected only 16 pairs in Patient 12 (N = 8) versus 17 at k = 1.3, illustrating that fixed distances cannot fully capture the adjacency graph when lattice spacing is heterogeneous.

## A.2.5.3 Convergence of Pair Count and PVDR_median_

Figure A3 summarizes the sensitivity analysis results in two panels. Panel (A) shows the number of adjacent sphere pairs as a function of k for all 29 configurations (faint gray lines), together with the mean ± SD (blue line with circles). Panel (B) shows the corresponding PVDR_median_ trajectories, with individual configurations in faint gray and the mean ± SD in green line with squares. The red dashed line in both panels marks k = 1.3.

In Panel (A), the per-configuration pair count trajectories rise steeply at low k values and flatten into a saturation plateau beyond k = 1.3, consistent with the saturation behavior reported in A.2.5.1 (mean: 17.69 pairs at k = 1.3; marginal gain +0.03 pairs from k = 1.3 to 1.4). In Panel (B), the PVDR_median_ estimate stabilizes at k = 1.3 (mean: 3.10 ± 0.61), with relative changes < 0.1% for all k ≥ 1.3. The convergence of both metrics at k = 1.3 — pair count saturating and PVDR_median_ stabilizing — jointly supports this coefficient as the value yielding robust and stable adjacency detection.


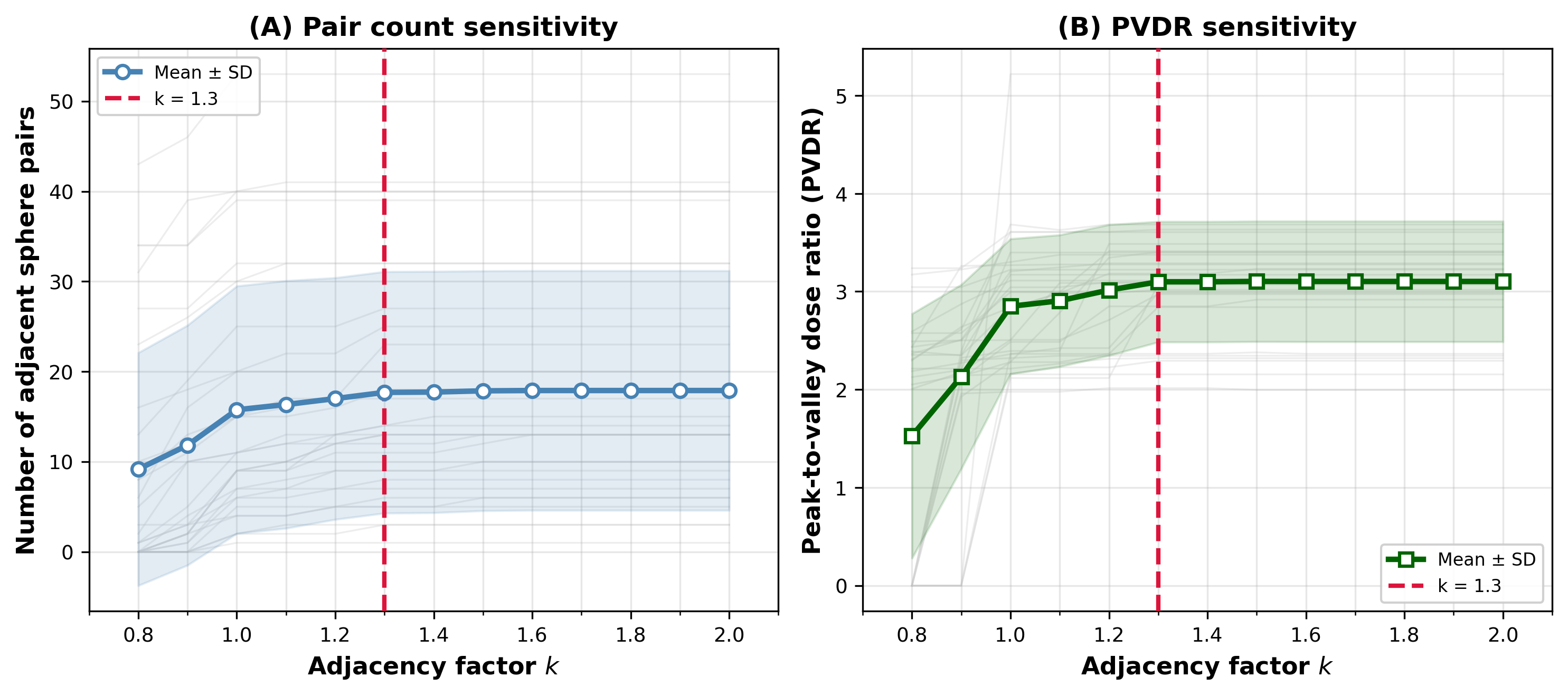


**Fig. A3.** Sensitivity analysis overview. (A) Pair count sensitivity: per-configuration trajectories (faint gray lines, n = 29) and mean ± 1 SD (blue line with circles). (B) PVDR sensitivity: per-configuration trajectories (faint gray lines) and mean ± 1 SD (green line with squares). The red dashed line marks k = 1.3 in both panels.

# A.3 Local Adjacency Algorithm and Global/Local Comparison

## A.3.1 Local Algorithm (Gabriel Graph)

Local mode constructs the adjacency graph using the Gabriel graph, a geometric structure that encodes local neighborhood relationships without global parameters. A vertex pair (i, j) is adjacent if and only if no third vertex k exists such that $d_{ik}^{2}+d_{jk}^{2}<d_{ij}^{2}$ (equivalently, the open sphere with diameter [i,j] contains no other vertex centroids). This criterion admits only 'direct' neighbors into the adjacency graph.

The geometric basis of the Gabriel graph criterion is illustrated in Fig. A4. Panel (A) depicts a rejection case in which a third vertex lies inside the sphere with diameter [i,j], whereas Panel (B) shows an acceptance case where no such interfering vertex exists.


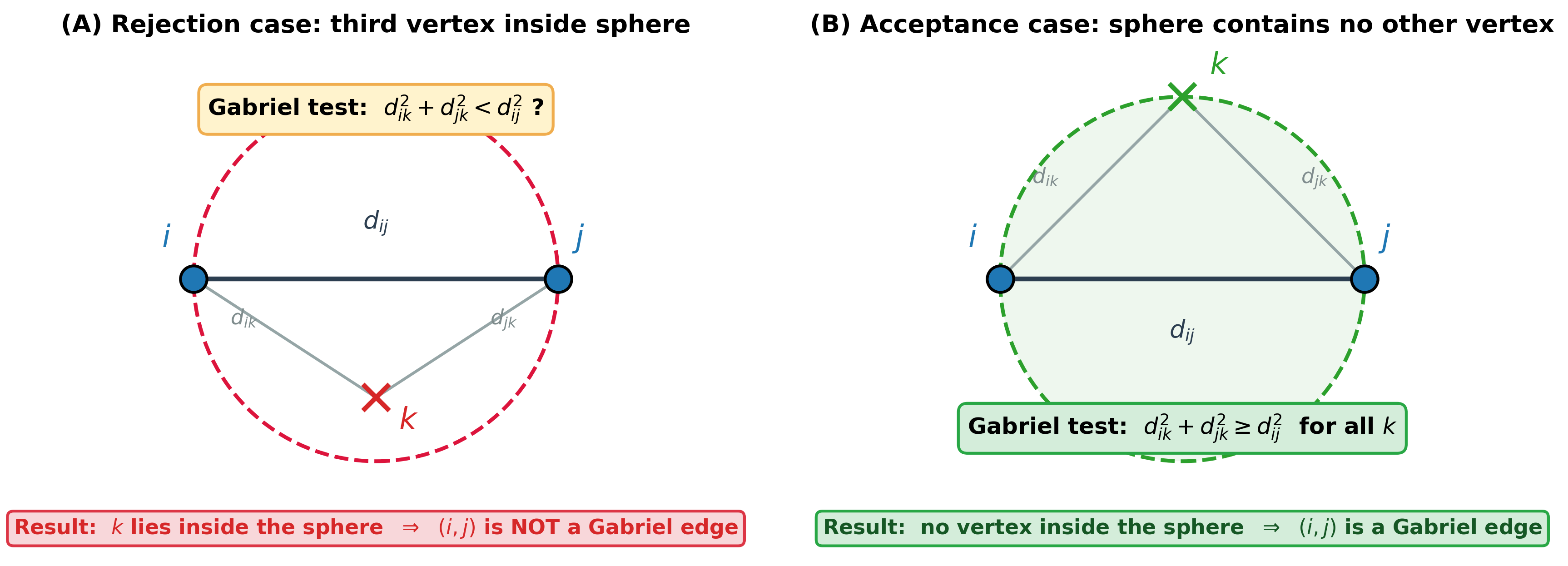


Fig. A4. Illustration of the Gabriel graph criterion. (A) Rejection case: a third vertex k lies inside the sphere with diameter d_ij, so the pair (i, j) is not considered adjacent. (B) Acceptance case: no other vertex lies inside the sphere, so (i, j) is accepted as a Gabriel edge.

Implementation proceeds in three steps: (1) computation of the n × n distance matrix, (2) testing of the Gabriel condition for each candidate pair, and (3) computation of PVDR_median_Local using the same dosimetric workflow as Global mode.

Global mode evaluates adjacency against a population-level threshold (k × D_median_), whereas Local mode evaluates each pair within its local geometric context using the Gabriel criterion, requiring no adjustable parameters. For any configuration, the Gabriel graph is uniquely determined by vertex geometry.

The two modes diverge at large N. In Global mode, increasing N inflates D_median_ and thus T_theory, potentially admitting face-diagonal connections (~42 mm). The 45 mm upper bound prevents body-diagonal inclusion at very large N. In contrast, the Gabriel criterion depends only on local relative distances and is unaffected by distant vertices.

## A.3.2 Global vs Local Concordance Analysis

Pearson correlation between Global and Local PVDR_median_ was r = 0.90 for the standard method and r = 0.88 for the optimized variant (both p < 0.001), indicating that both modes capture the same underlying dose distribution characteristic.

Divergence depends strongly on N (Fig. A5). For N ≤ 6, Pair Count was identical in all 14 configurations, and PVDR_median_ was concordant in 13 of 14; the single exception was Patient 12 (N = 4, asymmetric geometry: Global 3.41 vs Local 2.99). For small vertex populations with typical CTC spacing, no third vertex exists to violate the Gabriel condition for nearest-neighbor pairs, so the two graphs select the same edges. All 12 cases in the main manuscript cohort (N = 2–8) showed high concordance under both modes.


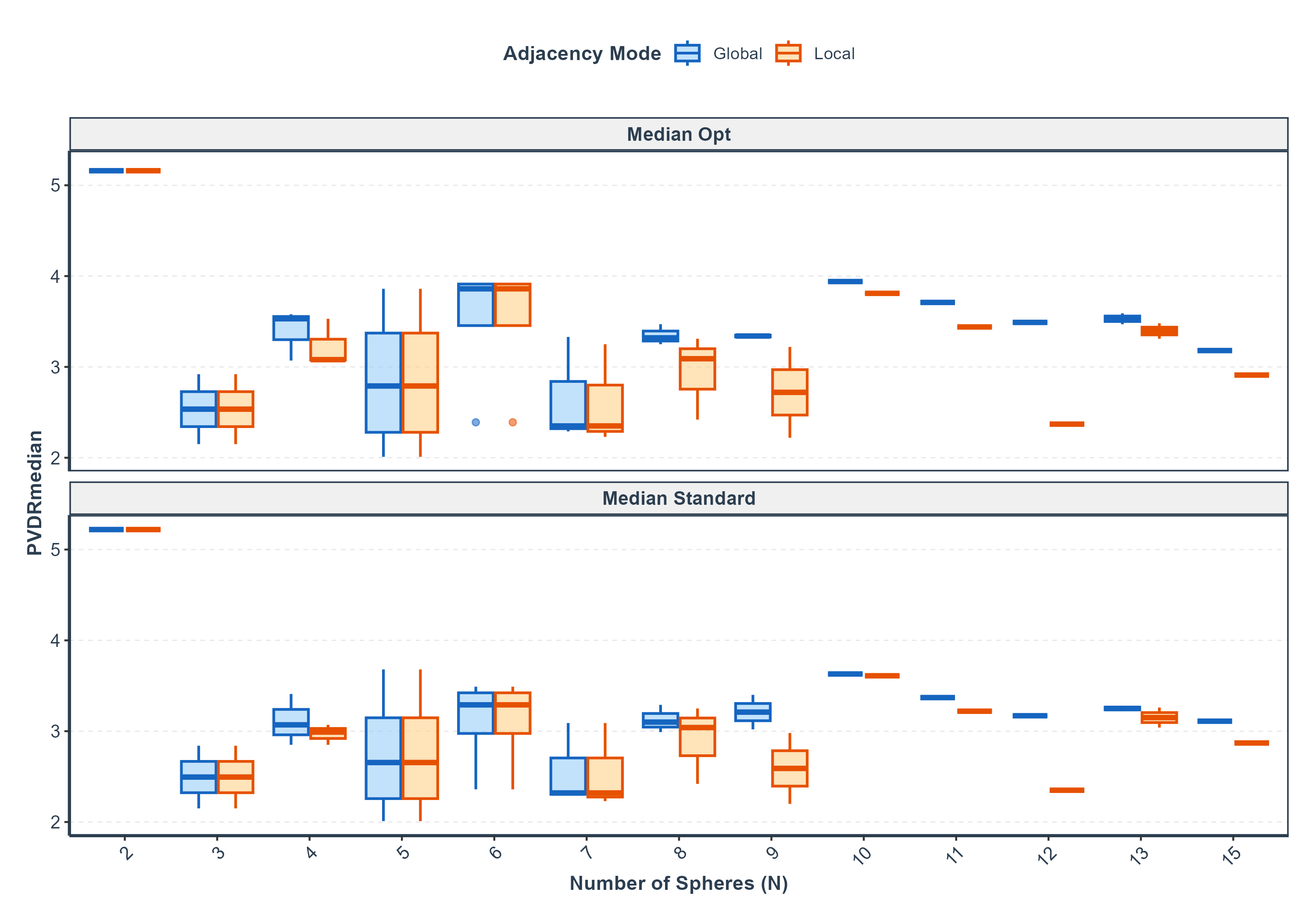


**Fig. A5.** Global vs Local PVDR_median_ stratified by vertex count (N). Upper panel: Optimized method; lower panel: Standard method. For N ≤ 6 the two modes are nearly concordant; divergence becomes systematic at N ≥ 7.

For N ≥ 7, Local mode consistently yields lower PVDR_median_ because it excludes face-diagonal connections captured by the Global threshold. The divergence increases with N: median differences of 0.2–0.3 at N = 8–9 and 0.3–0.5 at N = 13–15. The Optimized method shows the same pattern with slightly larger absolute values.

Local mode detected approximately 10–15% fewer pairs than Global mode (k = 1.3) for N ≥ 7, primarily because face-diagonal connections within the 45 mm bound violate the Gabriel criterion.

# A.4 Summary and Clinical Recommendations

The analyses presented in this Appendix address two independent questions about the adjacency algorithm and converge on practical, evidence-based recommendations.

(1) Robustness of k = 1.3: The number of detected adjacent pairs reaches a stable plateau at k = 1.3 (mean: 17.7 pairs), with marginal gains below 2% for all higher k values. PVDR_median_ simultaneously converges (mean: 3.10 ± 0.61, relative changes < 0.1% for k ≥ 1.3). Compared with the commonly used fixed 30 mm threshold, k = 1.3 detects 53% more pairs while adapting to patient-specific lattice spacing.

(2) Global/Local equivalence for small lattices: For N ≤ 6, the two modes produce identical Pair Count and concordant PVDR_median_ (13/14, ~93%). Either mode may be used for typical clinical lattice sizes.

(3) Global/Local divergence for large lattices: For N ≥ 7, Local mode detects fewer adjacent pairs than Global mode (approximately 10–15%). This does not indicate that one mode is correct and the other erroneous; rather, the two modes operationalize adjacency differently. The strong correlation (r ≥ 0.88) preserves clinical rankings, but absolute PVDR_median_ values are lower under Local mode because its adjacency graph is sparser. Neither mode is universally preferable: Global yields higher PVDR values by including face-diagonal connections, while Local yields lower PVDR values by excluding them.

(4) Practical recommendation: For N ≤ 6, either mode may be reported. For N ≥ 7, we recommend reporting both values to enable transparent comparison. If a single value is required, Local mode offers a parameter-free alternative grounded in geometric neighborhood relationships.

We recommend k = 1.3 as the default for Global mode in typical clinical lattice geometries (~3 cm CTC spacing). Users do not need to calibrate k themselves; k = 1.3 is preconfigured as the validated default. Future versions may expose this parameter for atypical geometries, pending further validation. Together with the parameter-free Local mode, these evidence-based options provide robust adjacency identification across the range of clinical lattice configurations.
